# Role of ATF3 as a prognostic biomarker and correlation of ATF3 expression with macrophage infiltration in hepatocellular carcinoma

**DOI:** 10.1186/s12920-020-00852-4

**Published:** 2021-01-06

**Authors:** Lijuan Li, Shaohua Song, Xiaoling Fang, Donglin Cao

**Affiliations:** Department of Laboratory Medicine, Guangdong Second Provincial General Hospital, No. 466 Xingang Middle Road, Haizhu District, Guangzhou, 510317 Guangdong Province China

**Keywords:** ATF3, Hepatocellular carcinoma, Database mining, Prognostic value, Functional network analysis

## Abstract

**Background:**

The abnormal expression of activating transcription factor 3 (ATF3), a member of the basic leucine zipper (bZIP) family of transcription factors, is associated with carcinogenesis. However, the expression pattern and exact role of ATF3 in the development and progression of hepatocellular carcinoma (HCC) remain unclear.

**Methods:**

We used UALCAN, ONCOMINE, Kaplan–Meier plotter, and cBioPortal databases to investigate the prognostic value of ATF3 expression in HCC.

**Results:**

ATF3 was found to be expressed at low levels in multiple HCC tumor tissues. Moreover, low ATF3 expression was significantly associated with clinical cancer stage and pathological tumor grade in patients with HCC. Therefore, low expression of *ATF3* was significantly associated with poor overall survival in patients with HCC. Functional network analysis showed that ATF3 regulates cytokine receptors and signaling pathways via various cancer-related kinases, miRNAs, and transcription factors. ATF3 expression was found to be correlated with macrophage infiltration levels and with macrophage immune marker sets in HCC patients.

**Conclusions:**

Using data mining methods, we clarified the role of ATF3 expression and related regulatory networks in HCC, laying a foundation for further functional research. Future research will validate our findings and establish clinical applications of ATF3 in the diagnosis and treatment of HCC.

## Background

Hepatocellular carcinoma (HCC) is the predominant type of liver cancer, the leading cause of cancer deaths worldwide, and the fourth most commonly diagnosed cancer in China [1, 2]. The global incidence of HCC is increasing and it has been predicted to exceed one million cases annually by 2025 [3]. Due to the high recurrence and metastasis rates of liver cancer, the 5-year survival rate of patients with advanced liver cancer does not exceed 5% [4]. Pathogenesis of HCC is very complex, involving environmental factors and various signal transduction pathways, reflecting multiple functions and interactions between genes at multiple steps [5]. Despite efforts to understand the mechanisms by which HCC develops, its molecular characteristics remain unclear. A comprehensive understanding of the pathogenesis and etiology of HCC would provide a basis for the development of prognostic and diagnostic biomarkers and therapeutic strategies.

Activating transcription factor 3 (ATF3) is a member of the ATF/cyclic AMP response element-binding (ATF/CREB) transcription factors family [6]. ATF3 plays roles in the modulation of stress and inflammatory responses, among other functions [7]. Additionally, ATF3 is a hub in the cellular adaptive response network [8]. However, ATF3 plays different roles in cancer development depending on the cancer cell type and environment. For example, ATF3 has been shown to promote or suppress apoptosis and cell proliferation, which are critical processes for cancer progression [6]. Furthermore, ATF3 has been shown to act as a tumor suppressor or oncogene in xenograft models using different cell lines [9–11]. Overexpression of ATF3 protects human breast cancer cells (malignant MCF10CA1a) from apoptosis and promotes metastasis. Conversely, ATF3 also increases apoptosis in the untransformed mammary epithelial cell line (MCF10A). These studies clearly indicate the dual functions of ATF3 in both oncogenesis and tumor suppression [12]. Chen et al. found that ATF3 exerts anti-cancer effect by regulating CYR61 in HCC [13]. Moreover, it has been reported that low levels of ATF3 may suppress hepatocellular oncogenesis [14]. These results reveal that *ATF3* is a novel tumor suppressor gene in HCC.

Advances in microarray and RNA-sequencing technology have contributed to the generation of substantial data in biological and biomedical research [15]. We studied public databases for data on ATF3 expression and mutation in patients with HCC. Using multi-dimensional analysis methods, we evaluated the molecular basis of HCC and the relationships between ATF3 expression and HCC pathogenesis and progression.

## Methods

### ONCOMINE

ONCOMINE (http://oncomine.org) is an online microarray database containing 715 datasets and 86,733 samples [16]. *ATF3* mRNA expression data were obtained from ONCOMINE using the following thresholds: *P* < 1e−04; fold-change = 2; data type, mRNA.

### UALCAN

UALCAN (http://ualcan.path.uab.edu) is an interactive portal that is used to analyze TCGA gene expression data [17]. In our study, UALCAN was used to analyze *ATF3* mRNA expression in primary HCC tissues and the association of *ATF3* mRNA expression with clinicopathologic parameters in HCC. P < 0.05 was considered statistically significant (*P < 0.05, **P < 0.01, ***P < 0.001).

### Human protein atlas

Human Protein Atlas (https://www.proteinatlas.org) includes immunohistochemistry-based expression data [18]. Direct comparisons of ATF3 protein expression between normal and HCC tissues were performed using immunohistochemical image analysis.

### Kaplan–Meier plotter

Kaplan–Meier plotter (http://kmplot.com/analysis/) was used to analyze the effect of ATF3 mRNA expression level on the prognosis of liver cancer. The OS, RFS, PFS, and DSS of HCC patients were determined by dividing the patients into high and low median expression groups. Kaplan–Meier survival chart was generated and 95% confidence interval risk rate and log rank p value [19].

### cBio portal

The cBio Cancer Genomics Portal (http://cbioportal.org) contains a multidimensional cancer genome dataset [20]. In this study, cBioPortal was used to analyze ATF3 alterations in TCGA LIHC samples. Mutations, CNVs, and mRNA expression data were obtained. The OncoPrint tab in the user interface was used to obtain an overview of genetic alterations in *ATF3*. ATF3 mutations and their relationship to OS and disease-free survival (DFS) in HCC patients were determined through Kaplan–Meier plots and log-rank tests were used to assess differences between survival curves.

### LinkedOmics

LinkedOmics (http://linkedomics.org) includes 32 TCGA cancer-associated multi-dimensional datasets [21]. LinkFinder module was used to research differentially expressed genes associated with ATF3 levels in the TCGA LIHC cohort (n = 371). The results were analyzed using Pearson’s correlation coefficient. All results are graphically represented in a volcano, heat map. We signed and sorted the LinkFinder results, and used GSEA to analyze GO terminology (biological process), KEGG pathway, kinase-target enrichment, miRNA-Target enrichment, and transcription factor-target enrichment [22]. Grade standard FDR < 0.05, simulated 500 times.

### TIMER

TIMER (https://cistrome.shinyapps.io/timer/) is an extensive resource for analyzing immune cell infiltration of different cancer types in TCGA [23]. TIMER infers abundance of tumor infiltrating immune cells (TIICs) from gene expression profiles using previously published deconvolution method [24]. We analyzed ATF3 expression in LIHC and the correlations between ATF3 expression and abundance of multiple immune infiltrates cells as well as tumor purity. ATF3 is represented by genetic symbols on the x-axis, and related tumor infiltrating immune cell markers are represented by genetic symbols on the y-axis. Gene expression levels are expressed using log2rsem.

### HCCDB database analysis

HCCDB (http://lifeome.net/database/hccdb/home.html) is a HCC expression profile database containing 15 public HCC gene expression data sets [25]. HCCDB provides visualization of results of computational analysis, such as differential expression analysis, tissue-specific expression analysis, and tumor-specific expression analysis.

### qRT-PCR verification

Normal human hepatic cell line LO2, human hepatocellular carcinoma cell lines SMMC-7721, HepG2 and Hep3B were purchased from the Cell Bank of Type Culture Collection of Chinese Academy of Sciences (Shanghai, China). All cells were cultured in DMEM medium containing 10% fetal bovine serum and 1% penicillin–streptomycin at 37 °C with 5% CO_2_. Total RNAs were isolated by using TRIzol (Invitrogen) and reverse transcription was performed with 1 μg of total RNA using rimeScript RT reagent kits (TAKARA Biotechnology, Dalian, China) following with the manufacturer's instructions, respectively. SYBR Green PCR master mix was employed for mRNA quantification. GAPDH was used as a control gene, and primer sequences of ATF3 as follows: forward, 5′- CCTCTGCGCTGGAATCAGTC -3′, reverse, 5′-TTCTTTCTCGTCGCCTCTTTTT-3′.

### GEO database

We collected four gene expression profiles containing HCC and adjacent samples (GSE14520, GSE25097, GSE76427 and GSE121248) from GEO database (https://www.ncbi.nlm.nih.gov/geo/). The four datasets were performed on different platforms. Characteristics of datasets included were displayed in Table 1.

**Table 1:** Details of hepatocellular carcinoma studies and associated microarray datasets from gene expression Omnibus database

Dataset	Platform	Samples size (tumor/control)
GSE14520	GPL3921 [HT_HG-U133A] Affymetrix HT Human Genome U133A Array	445 (225/220)
GSE25097	GPL10687 Rosetta/Merck Human RSTA Affymetrix 1.0 microarray, Custom CDF	511 (268/243)
GSE76427	GPL10558 Illumina HumanHT-12 V4.0 expression beadchip	167 (115/52)
GSE121248	GPL570 (HG-U133_Plus_2) Affymetrix Human Genome U133 Plus 2.0 Array	107 (70/37)

## Results

### ATF3 expression in patients with HCC

**Fig. 1:**
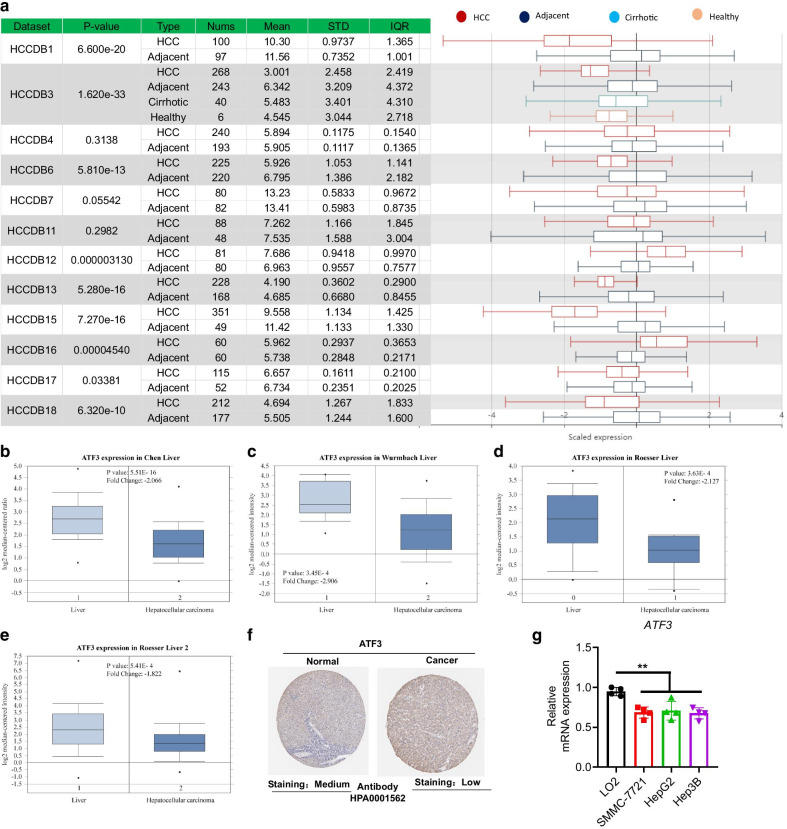
*ATF3* transcript levels in hepatocellular carcinoma (HCC). **a** Chart and plot showing the expression of ATF3 in tumor and adjacent normal tissues according to t-test in HCCDB. **b–e** Box plot showing *ATF3* mRNA levels in the Chen liver (**b**), Wurmbach liver (**c**), and Roessler liver datasets (**d**, **e**). **f** Representative immunohistochemical images of ATF3 in HCC and normal liver tissues (Human Protein Atlas). g RT-qPCR analysis showed ATF3 was downregulated in HCC cell lines compared with normal hepatocytes (n = 4). *P < 0.05; **P < 0.01

We analyzed the *ATF3* mRNA levels in tumor and corresponding normal tissues of different tumors types using ONCOMINE and TIMER. The results revealed that *ATF3* expression was lower in bladder urothelial carcinoma (BLCA), breast invasive carcinoma (BRCA), cholangiocarcinoma (CHOL), kidney chromophobe (KICH), and liver hepatocellular carcinoma (LIHC) cancer patients (Additional file 1: Fig. 1). Next, we evaluated *ATF3* transcription levels in multiple HCC studies from the HCCDB database, which includes 12 HCC cohorts. The expression of ATF3 mRNA was significantly lower in HCC tissues than in normal adjacent tissues (Fig. 1a). Furthermore, ONCOMINE revealed that ATF3 expression levels and DNA copy number variations (CNVs) were considerably lower in HCC tissues than in normal tissues. ATF3 exhibited lower expression levels in HCC tissues than in normal samples in the Chen liver dataset (fold change = − 2.066, *P* = 5.51E−16, Fig. 1b), Wurmbach liver dataset (fold change = −2.906, *P* = 3.45E−4, Fig. 1c), and Roesser liver dataset (Fig. 1d, 1e). In addition, we also detected substantially lower ATF3 mRNA in the livers of patients with HCC tumor tissues than in normal tissues using data from the Human Protein Atlas (Fig. 1f). Furthermore, we detected the expression of *ATF3* in HCC cell lines, and results showed that *ATF3* was significantly down-regulated in HCC cell lines compared to normal human hepatocyte (Fig. 1g). Finally, the expression of ATF3 was compared between HCC and adjacent samples in four GEO datasets and we found that significantly downregulated in HCC consistently (Table 1 and Additional file 1: Fig. 2). Taken together, our results demonstrated that ATF3 expression was lower in patients with HCC than in healthy controls at both the mRNA and protein levels.

### *ATF3* expression in subgroups of patients with HCC stratified by various criteria

**Fig. 2:**
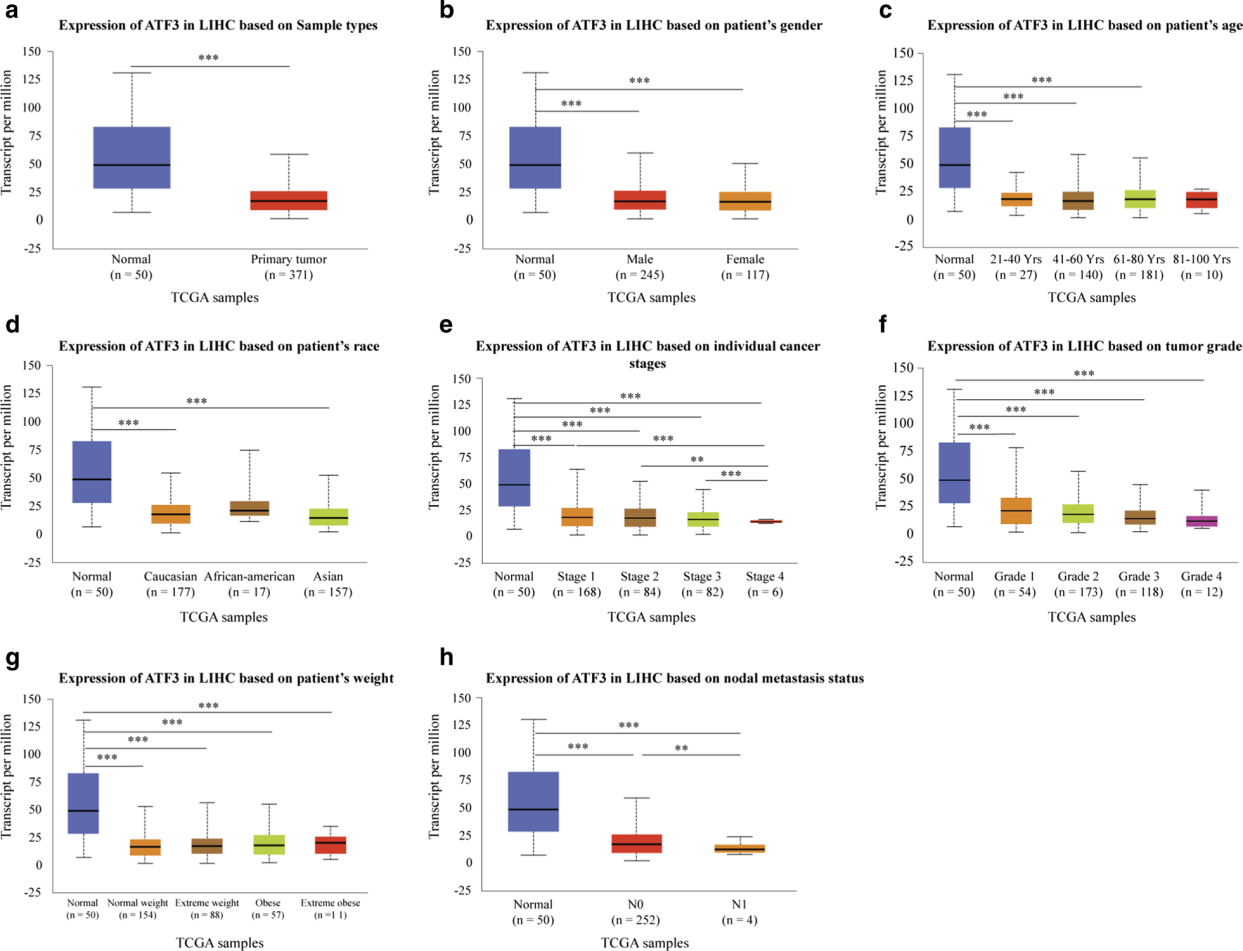
*ATF3* transcript levels in subgroups of HCC patients stratified by gender, age, and other criteria. **a** Boxplot showing relative expression levels of *ATF3* in normal and LIHC samples. **b** Boxplot showing relative expression levels of *ATF3* in healthy male or female individuals or male or female patients with LIHC. **c** Boxplot showing relative expression levels of *ATF3* in normal individuals of any age or in patients with LIHC aged 21–40, 41–60, 61–80, or 81–100 years. **d** Boxplot showing relative expression levels of *ATF3* in normal individuals of any ethnicity or in patients with LIHC of Caucasian, African-American, or Asian ethnicity. **e** Boxplot showing relative expression levels of *ATF3* in normal individuals or in patients with LIHC at stages 1, 2, 3, or 4. **f** Boxplot showing relative expression levels of *ATF3* in normal individuals or patients with LIHC with tumors classified as grade 1, 2, 3, or 4. **g** Boxplot showing relative expression levels of *ATF3* based on patient’s weight. **h** Boxplot showing relative expression levels of *ATF3* based on nodal metastasis status. *P < 0.05; **P < 0.01; ***P < 0.001

Sub-group analyses based on multiple clinicopathological features in 371 LIHC samples in TCGA consistently indicated lower transcription levels of ATF3 in HCC patients than in healthy controls (Fig. 2a). *ATF3* levels were substantially lower in patients with HCC than in healthy individuals of subgroups analyzed based on gender (Fig. 2b), age (Fig. 2c), ethnicity (Fig. 2d), disease stage (Fig. 2e), tumor grade (Fig. 2f), weight (Fig. 2g), and nodal metastasis (Fig. 2h). Thus, *ATF3* expression can be used as a diagnostic indicator in patients with HCC.

### Prognostic value of *ATF3* expression in patients with HCC

**Fig. 3:**
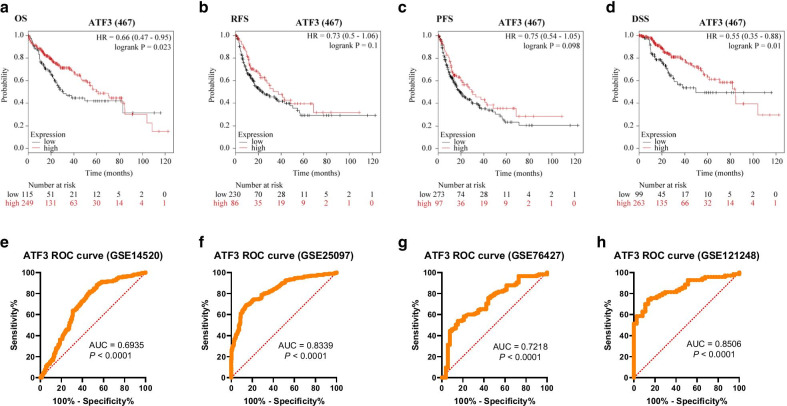
Prognostic value of ATF3 in HCC (Kaplan–Meier Plotter). Prognostic value of ATF3 in all patients with HCC based on **a** OS, **b** RFS, **c** PFS, and **d** DSS. OS, overall survival; RFS, relapse-free survival; PFS, progression-free survival; DSS, disease-specific survival. **e–h** ROC curve analysis of ATF3 in HCC

Prognostic value of *ATF3* mRNA expression in patients with liver cancer was determined using Kaplan–Meier plot. As shown in Fig. 3a, low levels of *ATF3* were associated with a shorter overall survival (OS) (HR = 0.66, *P* = 0.023). Recurrence-free survival (RFS) and progression-free survival (PFS) were not associated with *ATF3* mRNA expression levels in HCC (Fig. 3b–c). More importantly, low mRNA expression of *ATF3* was associated with a worse prognosis based on disease-specific survival (DSS) (Fig. 3d, HR = 0.55 and *P* = 0.01). These results indicated that *ATF3* expression might be an effective prognostic biomarker in HCC. We then used Kaplan–Meier plotter data to study the relationship between ATF3 expression and clinical characteristics of HCC patients. Low expression of *ATF* was associated with lower OS and PFS in male patients as well as in patients of Asian ethnicity (*P* < 0.05). Specifically, low *ATF3* mRNA expression was associated with shorter OS in stage 1/3/4 HCC, and was substantially associated with OS and PFS of grade 2 HCC (Table 2). Furthermore, low *ATF3* expression was associated with shorter OS in stage 1/2/3 of TNM category T (Table 2). More importantly, we found that low *ATF3* mRNA expression was correlated with lower OS and PFS after sorafenib treatment (Table 2), suggesting that *ATF3* is a novel predictive biomarker that could be used for evaluation of therapeutic HCC treatment. Low *ATF3* mRNA expression was also associated with lower OS and PFS after alcohol consumption and in patients without viral hepatitis infection (Table 2). Furthermore, ATF3 expression had a good performance in discriminating HCC across above four datasets (GSE14520: AUC = 0.6935, P < 0.0001; GSE25097: AUC = 0.8339, P < 0.0001; GSE76427: AUC = 0.7218, P < 0.0001 and GSE121248: AUC = 8506, P < 0.0001) (Fig. 3e–h). These results suggest that *ATF3* expression level substantially affects the progression and prognosis of HCC patients.

**Table 2:** Correlation of ATF3 mRNA expression and clinical prognosis in HCC with different clinicopathological factors

Clinicopathological characteristics	Overall survival (OS)			Progression-free survival (PFS)		
	N	Hazard ratio (HR)	P-value	N	Hazard ratio (HR)	P-value
Sex						
Female	118	1.71(0.98–2.98)	5.60E−02	121	1.44 (0.87–2.41)	1.60E−01
Male	246	0.52(0.33–0.83)	*3.90E−03*	250	0.61 (0.42–0.89)	*9.00E−03*
Race						
White	181	0.67(0.41–1.09)	1.10E−01	184	1.41 (0.85–2.32)	1.80E−01
Asian	155	0.43(0.24–0.77)	*3.80E−03*	157	0.54(0.33–0.88)	*1.20E−02*
Stage						
1	170	2(1.02–3.91)	*4.00E−02*	171	1.51 (0.9–02.52)	1.10E−01
1 + 2	253	0.69 (0.4–1.17)	1.60E−01	256	0.73 (0.48–1.12)	1.50E−01
2	83	0.49(0.21–1.14)	9.00E−02	84	0.53 (0.3–0.96)	*3.40E−02*
2 + 3	166	0.37(0.23–0.61)	*3.50E−05*	170	0.66 (0.44–1)	4.60E−02
3	83	0.32(0.18–0.6)	*1.50E−04*	85	0.81 (0.46–1.44)	4.80E−01
3 + 4	87	0.36(0.2–0.64)	*3.50E−04*	90	0.83 (0.48–1.45)	5.10E−01
4	4			5		
Grade						
1	55	0.49(0.18–1.35)	1.60E−01	55	0.76(0.31–1.89)	5.50E−01
2	174	0.3(0.18–0.52)	*4.30E−06*	177	0.540.34–0.86)	*7.80E−03*
3	128	0.31(0.71–2.42)	3.80E−01	121	1.2 (0.72–2)	4.90E−01
4	12			12		
AJCC stage T						
1	180	1.89(1.01–3.55)	*4.40E−02*	181	1.49(0.91–2.45)	1.10E−01
2	90	0.41(0.19–0.89)	*2.00E−02*	92	0.68 (0.38–1.2)	1.80E−01
3	78	0.32(0.17–0.6)	*1.90E−04*	80	0.79 (0.43–1.45)	4.40E−01
4	13			13		
Vascular invasion						
None	203	0.63(0.37–1.06)	8.10E−02	205	0.7 (0.43–1.15)	1.60E−01
Micro	90	0.46(0.17–1.21)	1.10E−01	90	0.46(0.17–1.21)	1.10E−01
Macro	16			16		
Sorafenib treatment						
Treated	29	0.28(0.08–0.95)	*3.00E−02*	30	0.25 (0.09–0.66)	*2.50E−03*
Alcohol consumption						
Yes	115	0.36(0.19–0.38)	*1.00E−03*	117	0.53 (0.29–0.94)	*2.80E−02*
No	202	0.64(0.37–1.1)	1.10E−01	205	0.83 (0.53–1.29)	4.10E−01
Hepatitis virus						
Yes	150	0.76(0.91–3.43)	9.10E−02	143	1.37 (0.87–2.18)	1.80E−01
None	167	0.59(0.38–0.94)	*2.30E−02*	169	0.49 (0.28–0.83)	*7.20E−03*

The italic values highlights statistically significant results *P* < 0.05

### Associations between *ATF3* mutations and survival in HCC

**Fig. 4:**
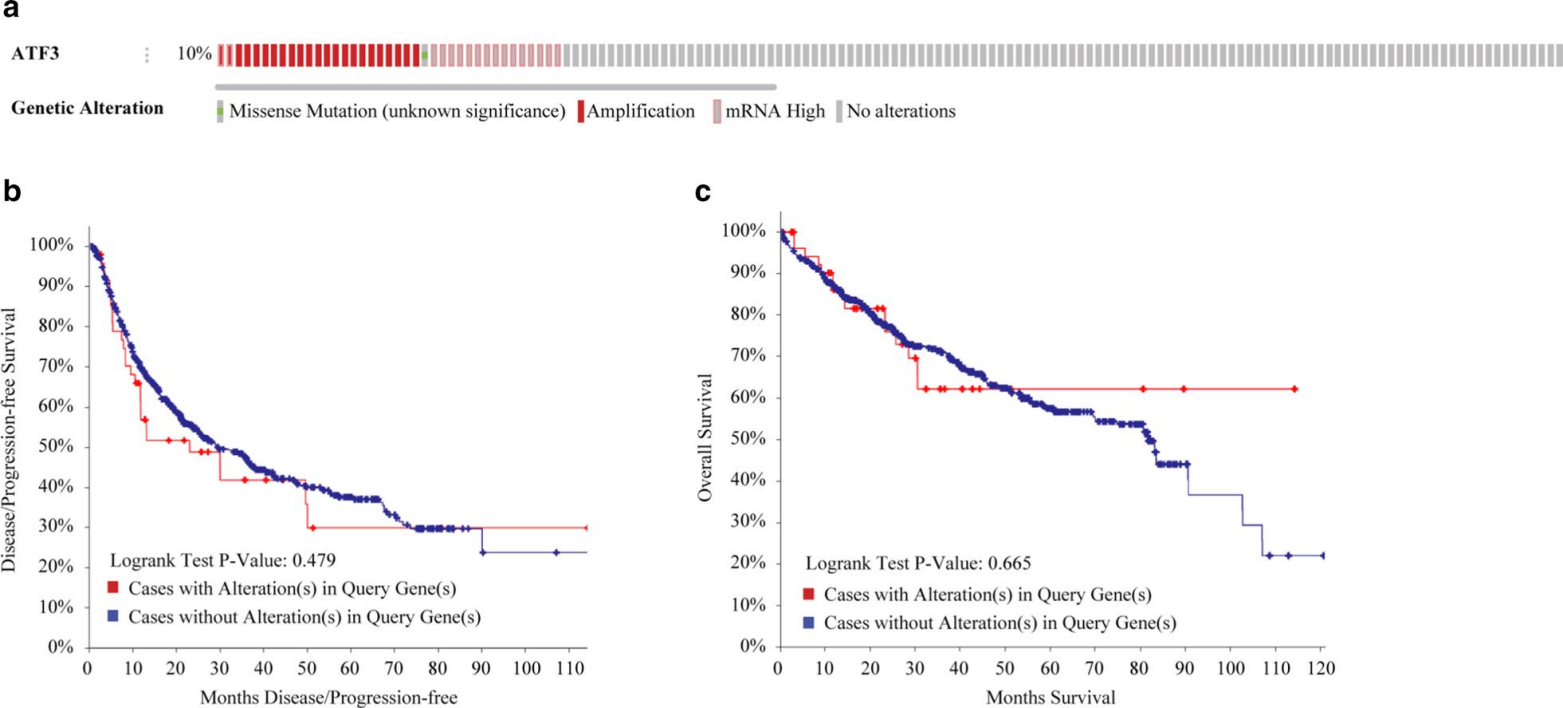
Mutation frequency and survival analysis of *ATF3* in HCC. **a** Visual summary of alterations in *ATF3* obtained using OncoPrint. Different mutation types are highlighted in different colors. **b** Kaplan–Meier estimates of overall survival in cases with or without *ATF3* alterations. **c** Disease/progression-free survival determined by the Kaplan–Meier method in cases with or without *ATF3* alterations

We used cBioPortal to evaluate genetic alterations in *ATF3* in HCC based on sequencing data of LIHC patients. *ATF3* was altered in 39 of 372 (10.4%) patients with LIHC (Fig. 4a), including various mutation types such as missense mutations and amplifications. However, Kaplan–Meier plot and log-rank tests revealed that genetic mutations in *ATF3* were not significantly associated with a lower OS (Fig. 4b, P = 0.665) or disease-free survival (DFS) (Fig. 4c, P = 0.479) in patients with HCC.

### Enrichment analysis of *ATF3* functional networks in HCC

**Fig. 5:**
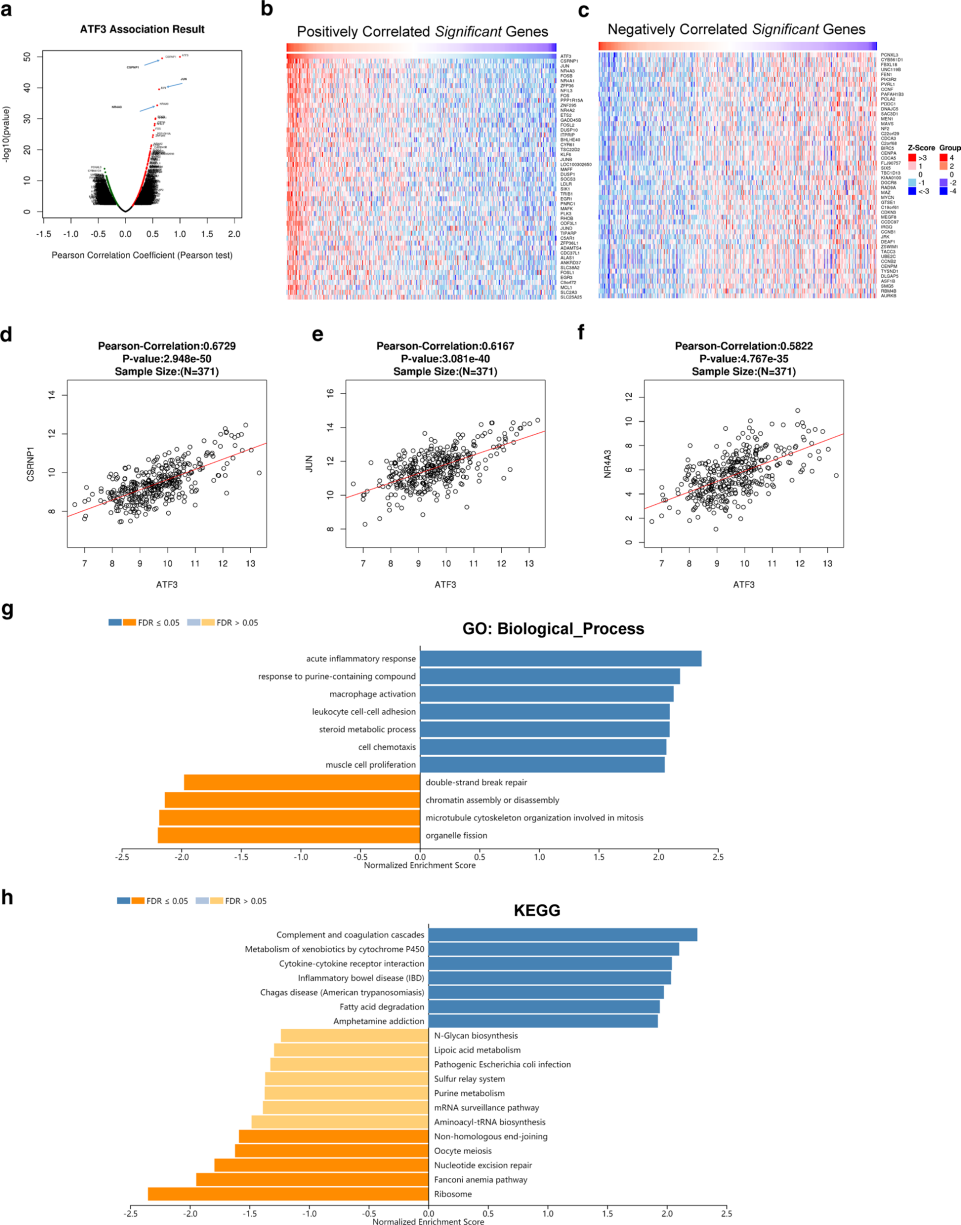
Differentially expressed genes correlated with *ATF3* in HCC. **a** Pearson correlation coefficients of relationships between *ATF3* and differentially expressed genes in LIHC. **b–c** Heat maps showing genes that are positively and negatively correlated with *ATF3* in LIHC (TOP 50). Red indicates positively correlated genes and green indicates negatively correlated genes. **d–f** Scatter plot showing Pearson correlation coefficients for the relationship between *ATF3* expression and *CSRNP1* (**d**), *JUN* (**e**), and *NR4A3* (**f**). **g–h** Significantly enriched GO annotations (**g**) and KEGG pathways (**h**) of ATF3 in LIHC cohort. Blue represents the LeadingEdgeNum, and orange represents the false discovery rate (FDR)

The Function module of LinkedOmics was used to analyze mRNA sequencing data from 371 patients with LIHC in TCGA. As shown in the volcano chart (Fig. 5a) and heat map (Fig. 5b, c), 50 gene sets were substantially positively or negatively correlated with *ATF3*. The results suggest that *ATF3* has a substantial effect on the transcriptome. *ATF3* expression showed strong positive association with expression of CSRNP1 (Pearson correlation coefficient = 0.6729, *P* = 2.948e−50), JUN (Pearson correlation coefficient = 0.6167, *P* = 3.081e−40), and NR4A3 (Pearson correlation coefficient = 0.5822, *P* = 4.767e−35), reflecting changes in the suppression of tumor growth (Fig. 5d–f). GO terms identified in gene set enrichment analysis (GSEA) revealed that differentially expressed genes correlated with *ATF3* were mainly involved in acute inflammatory response and macrophage activation (Fig. 5g). KEGG pathway analysis showed enrichment of complement and coagulation cascade pathways and cytokine-cytokine receptor interactions, which regulate cancer-related signaling pathways (Fig. 5h).

### ATF3 networks involve kinases, miRNAs, and transcription factors in HCC

To reveal the targets networks of *ATF3* in HCC, we analyzed positively correlated gene sets generated by GSEA, such as kinases, miRNAs, and transcription factor. The top 5 most significant kinase networks were related to mitogen-activated protein kinase, inhibitor of nuclear factor kappa B kinase subunit beta (Kinase_IKBKB), cyclin-dependent kinase 3 (Kinase_CDK3), glycogen synthase kinase 3 beta (Kinase_GSK3B), and FER tyrosine kinase (Kinase_FER) (Table 3). The miRNA-target network was associated with (ATTACAT) MIR-380-3P, (TGAATGT) MIR-181A, (TATTATA) MIR-374, (ACTTTAT) MIR-142-5P, and (TAATGTG) MIR-323 (Table 3). Transcription factor-target network was mainly related to CREB family members, including ATF3_Q6, CREBP1_Q2, ATF_01, CREB_Q2_01, and CREB_Q2 (Table 3). In fact, low expression of these kinase genes and transcription factors was considerably associated with worse OS prognosis in patients with HCC (Additional file 1: Fig. 3).

**Table 3:** Kinase, miRNA, and transcription factor targets of ATF3 in HCC

Category	Gene set	LeadingEdgeNum	FDR
Kinase	Kinase_MAPK8	76	0
	Kinase_IKBKB	9	0.040725
	Kinase_CDK3	7	0.084733
	Kinase_GSK3B	14	0.085654
	Kinase_FER	8	0.079594
miRNA	ATTACAT, MIR-380-3P	24	0
	TGAATGT, MIR-181A, MIR-181B, MIR-181C, MIR-181D	93	0.0028376
	TATTATA, MIR-374	54	0.0028376
	ACTTTAT, MIR-142-5P	62	0.0033105
	TAATGTG, MIR-323	43	0.0035470
Transcription Factor	V$ATF3_Q6	38	0
	V$CREBP1_Q2	53	0
	V$ATF_01	34	0
	V$CREB_Q2_01	31	0
	V$CREB_Q2	34	0

LeadingEdgeNum, number of leading edge genes; FDR, false discovery rate from Benjamini and Hochberg tests from a gene set enrichment analysis (GSEA). V$, annotation in the Molecular Signatures Database (MSigDB) for transcription factors (TF)

### ATF3 is correlated with tumor purity and immune infiltration level in HCC

**Fig. 6:**
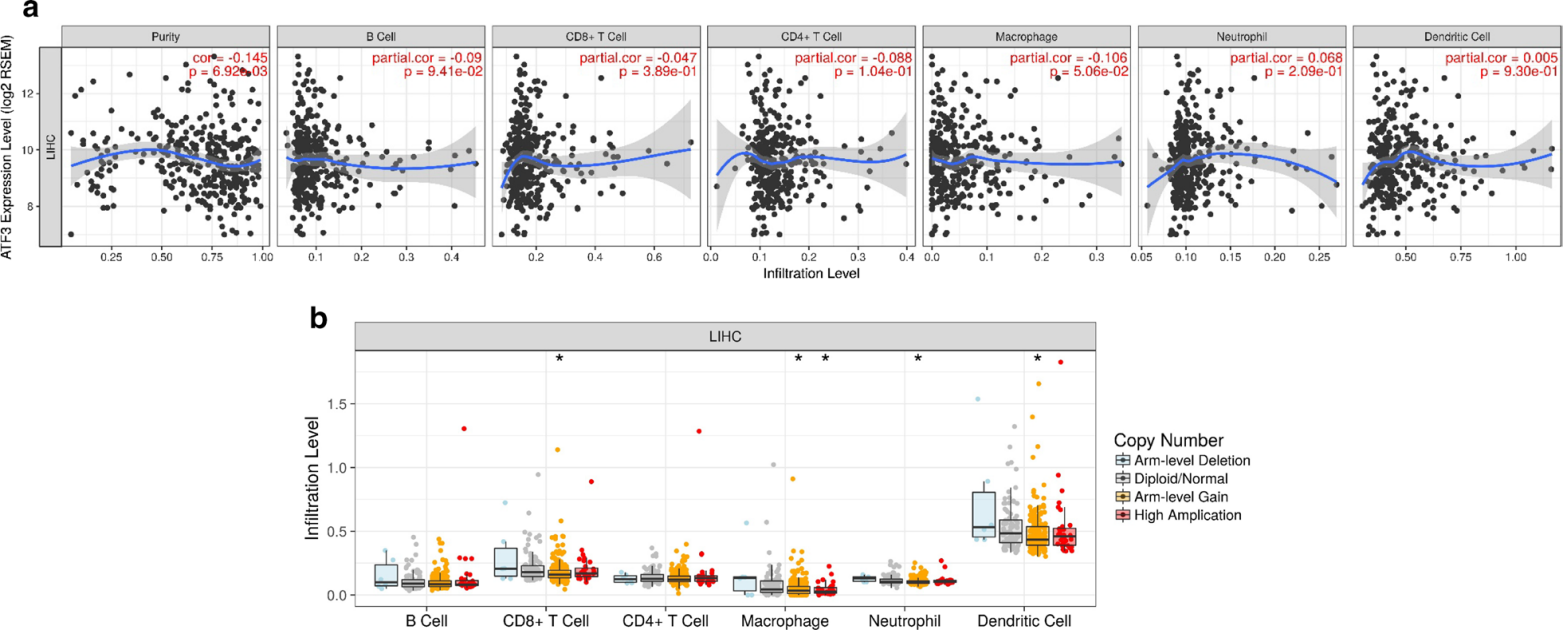
Correlations between ATF3 expression and immune infiltration level in HCC. (**a**) ATF3 expression relates to tumor purity and correlates with macrophage infiltration levels in LIHC. (**b**) ATF3 copy number variable (CNV) affects infiltration levels of CD8 + T cells, macrophages, neutrophils, and dendritic cells in HCC. *P < 0.05; **P < 0.01; ***P < 0.001

**Fig. 7:**
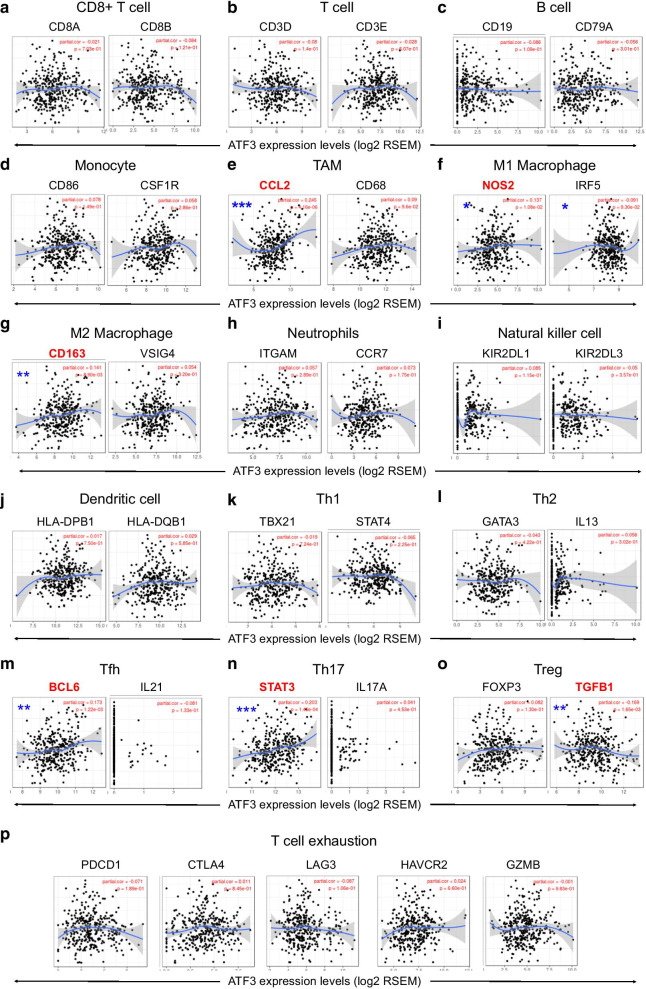
Correlation of ATF3 expression with markers of immune cells in HCC. Correlations between ATF3 and **a** CD8 + T cell markers (CD8A and CD8B); **b** T cell (general) markers (CD3D and CD3E); **c** B cell markers (CD19 and CD79A); **d** Monocyte markers (CD86 and CSF1R); **e** TAM markers (CCL2 and CD68); **f** M1 macrophage markers (NOS2 and IRF5); **g** M2 macrophage markers (CD163 and VSIG4); **h** Neutrophil markers (ITGAM and CCR7); **i** NK cell markers (KIR2DL1 and KIR2DL3); **j** Dendritic cell markers (HLA-DPB1 and HLA-DQB1); **k** Th1 cell markers (TBX21 and STAT4); **l** Th2 cell markers (GATA3 and IL13); **m** Tfh cell markers (BCL6 and IL21); **n** Th17 cell markers (STAT3 and IL17A); **o** Treg cell markers (Foxp3 and TGFB1); **p** T cell exhaustion markers (PDCD1, CTLA4, LAG3, and GZMB). Purity, correlation adjusted by purity. *P < 0.01; **P < 0.001; ***P < 0.0001

Tumor infiltrating lymphocytes (TIL) are independent predictors of cancer survival [26]. Therefore, we used TIMER to study whether the expression of ATF3 is related to the level of infiltrating lymphocytes in liver cancer. ATF3 expression exhibited significant negative correlation with tumor purity (r = − 0.145, *P* = 6.92E−03) and slight association with dominant macrophage levels (Fig. 6a). Specifically, ATF3 CNV was significantly associated with infiltration levels of CD8 + T cells, macrophages, neutrophils, and dendritic cells (Fig. 6b). In addition, in order to expand the understanding of the interaction between ATF3 and immune markers, we analyzed the correlation between ATF3 expression and various immune markers, including immune marker genes for tumor infiltrating lymphocytes (TILs) and immune suppression and immune checkpoint gene sets (Fig. 7a–p). After adjusting for tumor purity, the results showed that ATF3 expression was significantly correlated with macrophage subpopulations, including tumor-associated macrophages (TAMs; CCL2, r = 0.245, *P* = 4.16e−06), M1 (NOS2, r = 0.137, *P* = 0.08e−02), and M2 (CD163, r = 0.141, *P* = 8.90e−03) (Fig. 7e–g). The results suggested that expression of ATF3 is related to the macrophage subset of immune infiltration in HCC.

## Discussion

Abundant data are available regarding the dual roles of ATF3 in the protection of both normal and cancer cells from further transformation and in the promotion of tumor progression [27]. We used bioinformatics methods to analyze published sequencing data to understand the function of ATF3 in HCC and its regulatory network in detail, and thus, guide future studies on HCC and identification of novel biomarkers.

Using ONCOMINE and TIMER datasets, we found that ATF3 levels were down-regulated in human HCC and they were correlated with patient clinical characteristics. We found that low levels of *ATF3* were associated with lower OS and DFS in patients with HCC, suggesting that the gene acts as a tumor suppressor. These results were consistent with the results of a previous study that demonstrated low expression of ATF3 at the protein and mRNA levels in HCC [14]. Similarly, ATF3 is down-regulated in esophageal squamous cell carcinoma [28]. It has been reported that the expression of ATF3 is lower in human colorectal tumors than in normal adjacent tissues, while overexpression of ATF3 in vivo can reduce the volume of mouse tumor xenografts by 54% [29]. More importantly, higher ATF3 protein levels were detected in non-enveloped HCC patients, suggesting that ATF3 may be a target for migration inhibition [30]. In present study, low expression of ATF3 was correlated with poor prognosis of HCC in stage 1/3/4, T1-T3, and grade 2, with highest HR for low OS and PFS (Table 2). Taken together, these findings strongly suggest that ATF3 could be a biomarker for the prognosis of HCC.

Conflicting results regarding the expression and function of ATF3 in tumors can be explained by differences in ATF3 expression pattern among tumor types and cell lines. First, diverse signaling molecules and pathways may be involved. ATF3 can be induced by a variety of extracellular stress signals, including MAPK, P53, c-Myc, and TGF-β, which are involved in cell proliferation, differentiation, transformation, and death [31]. Our results suggest that the functional network involving ATF3 participates primarily in cytokine receptor signaling pathway activation. We found that ATF3 in HCC is related to the kinase network, including MAPK8, IKBKB, and CDK3. These kinases regulate genome stability and cell proliferation. Abnormal regulation of various transcription factors, such as NF-κB, AP-1, and Ets, is thought to play an important role in tumorigenesis, and each transcription factor can regulate multiple interacting signaling pathways. Through network analysis, it was predicted that part of the ATF3 transcription complex also contains members of the NF-κB family [32]. These transcription factor signaling pathways provide novel candidate targets for the prevention and treatment of HCC. Second, differences in the effects of ATF3 may be explained by differences in dimer partners. ATF3 can form a homodimer that inhibits transcription and a heterodimer complex with c-Jun or JunD that inhibits or activates target gene expression [33]. In addition, we found a marked positive association between ATF3 expression and NR4A3 in patients with HCC. In aggressive lymphoma, NR4A3 has powerful tumor suppressor function similar to NR4A1 [34, 35]. However, the relationship between NR4A3 and HCC warrants further study. Taken together, our results clearly reveal that ATF3 plays a role in inhibiting tumor growth in patients with HCC.

Using GSEA enrichment for analysis of the target gene set, we determined important target kinases, miRNAs, and transcription factor networks. We found that ATF3 in HCC was associated with a network of kinases, including MAPK, IKBKB, and CDK3. These kinases regulate tumor signaling pathways, NF-κB signaling, and the cell cycle [36–38]. Dysregulation of MAPK signaling pathways in HCC has been reported previously [36]. Our study also identified several miRNAs involved in transcriptional regulation and carcinogenesis. MiR-380-3p was shown to target SOX6 to regulate bactericidal effects by affecting β-catenin MITF transcription and translation, providing insights into the mechanisms by which miR-380-3p controls melanogenesis [39]. To further clarify genetic alterations, functions, and carcinogenic mechanisms of ATF3, we evaluated the frequency of *ATF3* mutations in HCC and obtained an estimate of 10% based on publicly available data. In addition, the OS and DFS of a case with a query genetic alteration of *ATF3* were lower than those without a query genetic alteration, but the differences were not statistically significant. Another important aspect of this study is that in HCC, ATF3 expression is correlated with TAMs and M1/M2 macrophage infiltration levels. Furthermore, gene markers of different macrophages such as CCL2, NOS2, and CD163 are correlated with ATF3 expression. These results suggest that ATF3 plays an important role in the recruitment of macrophages and in the regulation of immune infiltration in HCC.

Targeted gene analysis from public online database revealed multi-level evidence of the importance of ATF3 in liver cancer development and supported its role as a biomarker in HCC. In addition, ATF3 expression may be involved in the regulation of tumor-related and M1/M2 macrophages. Therefore, ATF3 may play an important role in immune cell infiltration and may serve as a biomarker for prognosis in patients with HCC. An obvious limitation of our study is that transcriptome sequencing can only detect static mutations and cannot directly provide information related to protein activity or expression levels; these issues should be addressed in subsequent research using molecular biology techniques.

## Conclusions

The findings of our study revealed the role of ATF3 as a biomarker in HCC. In addition, it was found that ATF3 expression potentially contributes to the regulation of tumor-associated and M1/M2 macrophages. Therefore, ATF3 likely plays an important role in immune cell infiltration and could be used as a prognostic biomarker in patients with HCC.

## Supplementary Information

**Additional file 1.** **Supplementary Figure 1.** Overall expression level of ATF3 in different tumor types.

## Data Availability

Direct web links of datasets about; Oncomine: http://oncomine.org; UALCAN: http://ualcan.path.uab.edu; The Human Protein Atlas: https://www.proteinatlas.org; cBioPortal: http://cbioportal.org; LinkedOmics: http://linkedomics.org; TIMER: https://cistrome.shinyapps.io/timer/; HCCDB: http://lifeome.net/database/hccdb/home.html; Transcriptome data was downloaded from NCBI Gene Expression Omnibus (GEO) profiles database (https://www.ncbi.nlm.nih.gov/geo/), accession number: GSE14520, GSE25097, GSE76427 and GSE121248. The datasets used and/or analyzed during the current study are available from the corresponding author on reasonable request.

## Abbreviations

ATF3: Activating transcription factor 3
TCGA: The cancer genome atlas
OS: Overall survival
RFS: Recurrence-free survival
PFS: Progression-free survival
DSS: Disease-specific survival
CNVs: Copy number variations
TIIC: Tumor-infiltrating immune cell
DFS: Disease-free survival
HCC: Hepatocellular carcinoma
GSEA: Gene set enrichment analysis
