# Supplementary material for: Role of ATF3 as a prognostic biomarker and correlation of ATF3 expression with macrophage infiltration in hepatocellular carcinoma
Source: BMC Med Genomics. 2021 Jan 6;14:8. doi: 10.1186/s12920-020-00852-4 (PMC7789720; doi:10.1186/s12920-020-00852-4)
Supplement: Supplementary file 1 — Additional file 1. Supplementary Figure 1. Overall expression level of ATF3 in different tumor types. [file 12920_2020_852_MOESM1_ESM.pptx]

## Slide 1
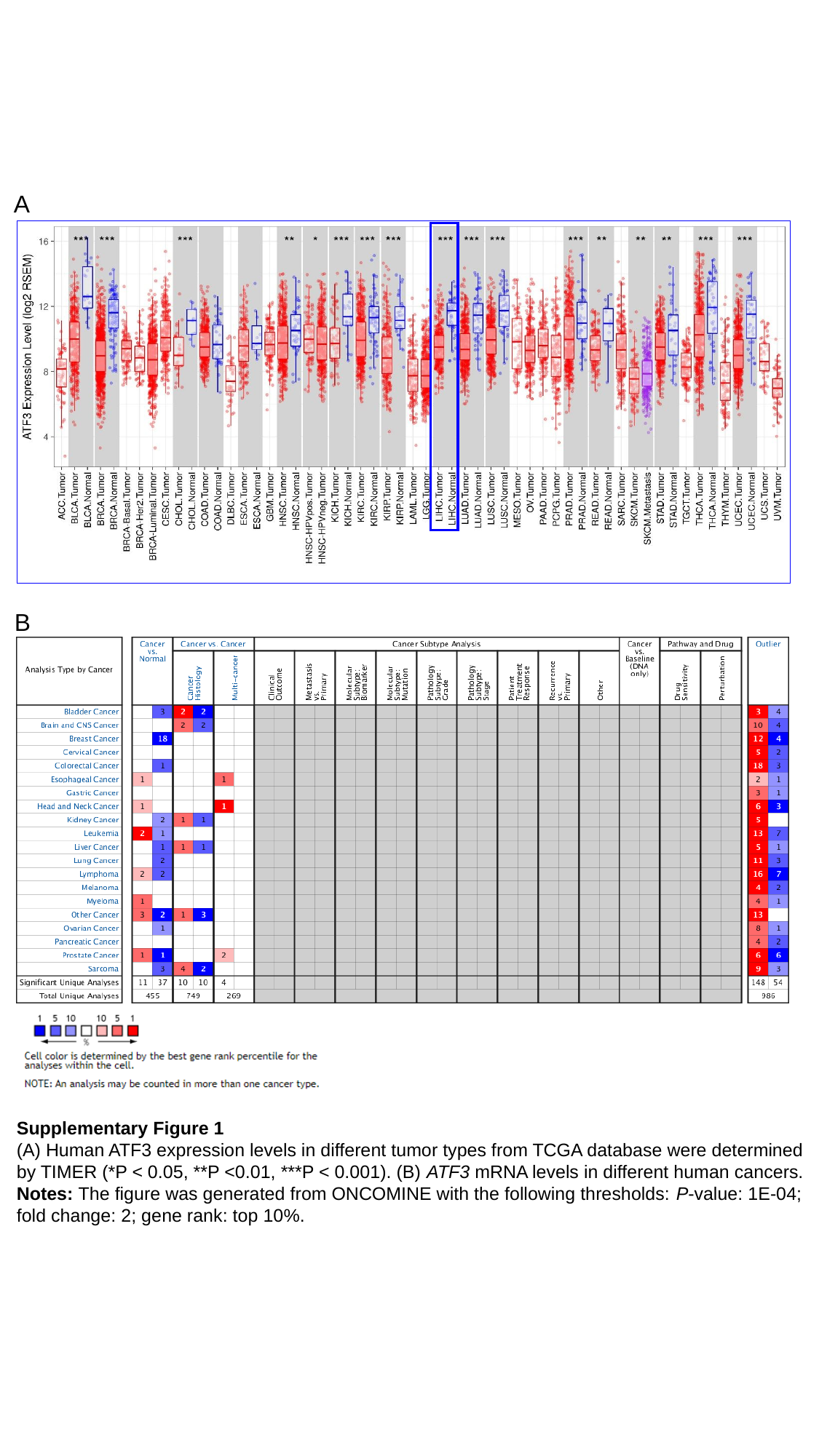

A
B
Supplementary Figure 1
(A) Human ATF3 expression levels in different tumor types from TCGA database were determined by TIMER (*P < 0.05, **P <0.01, ***P < 0.001). (B) ATF3 mRNA levels in different human cancers.
Notes: The figure was generated from ONCOMINE with the following thresholds: P-value: 1E-04; fold change: 2; gene rank: top 10%.

## Slide 2
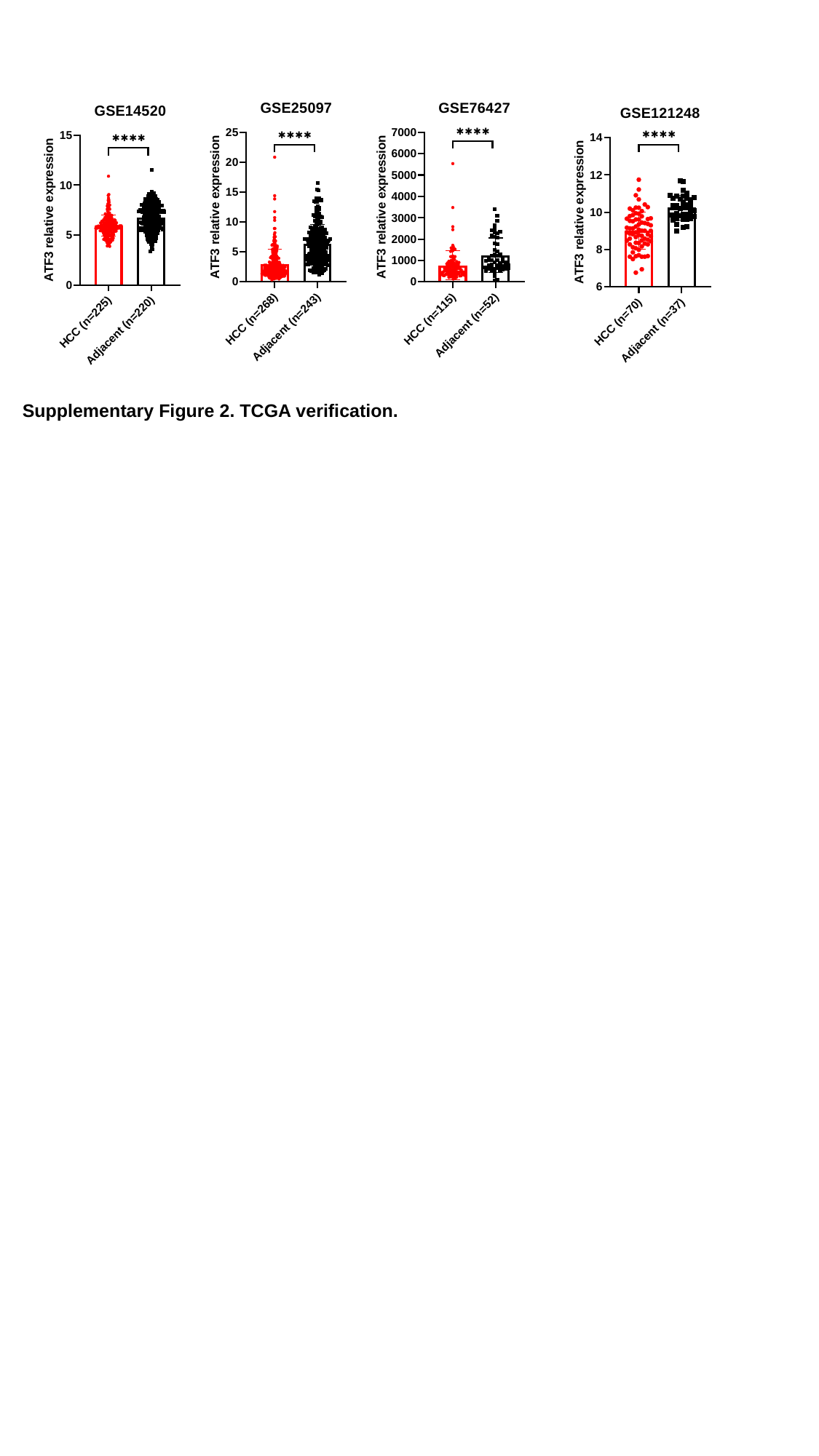

Supplementary Figure 2. TCGA verification.

## Slide 3
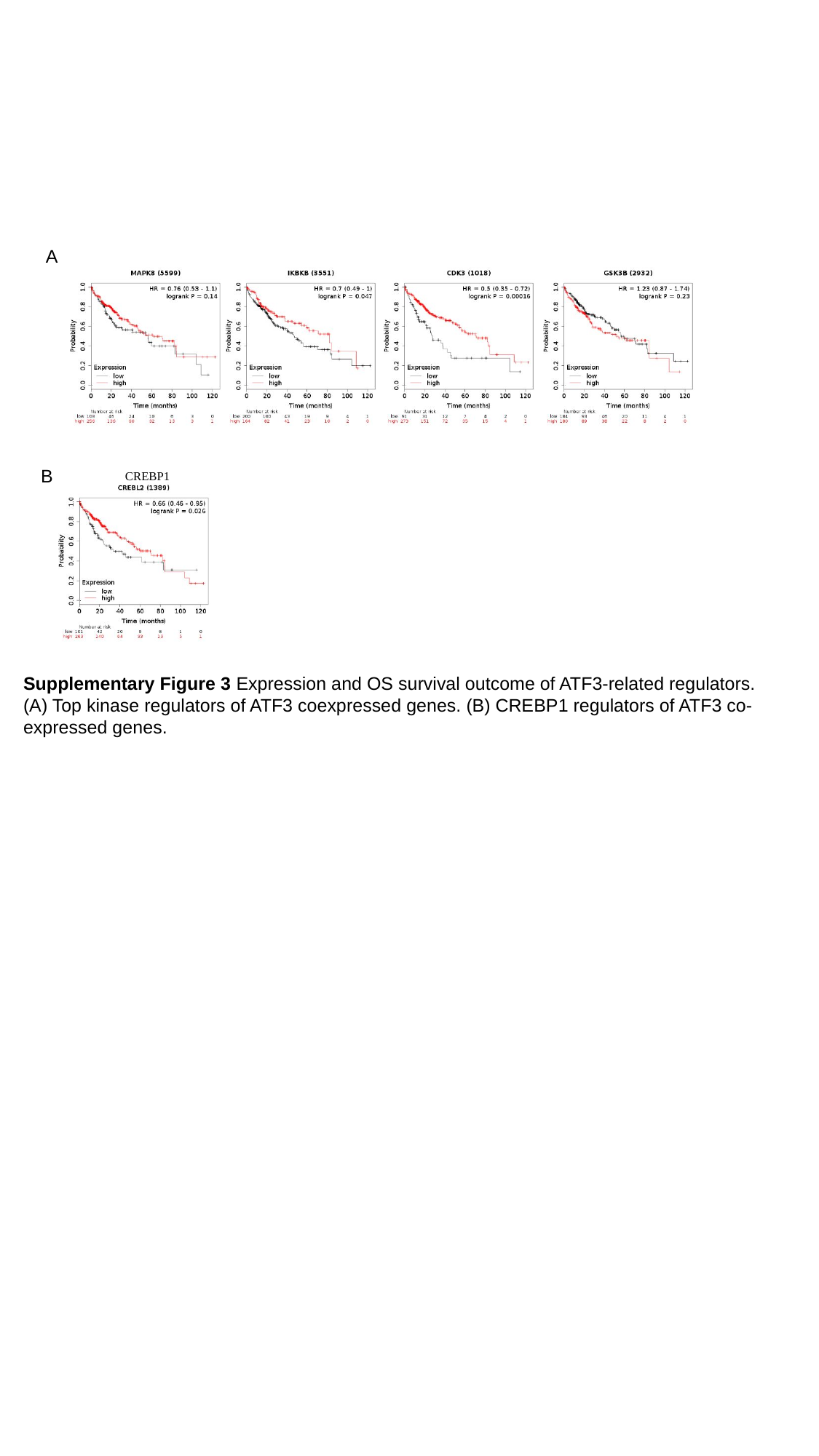

A
B
CREBP1
Supplementary Figure 3 Expression and OS survival outcome of ATF3-related regulators.
(A) Top kinase regulators of ATF3 coexpressed genes. (B) CREBP1 regulators of ATF3 co-expressed genes.
